# Factors influencing job preferences of health workers providing obstetric care: results from discrete choice experiments in Malawi, Mozambique and Tanzania

**DOI:** 10.1186/s12992-016-0222-4

**Published:** 2016-12-20

**Authors:** Eilish McAuliffe, Marie Galligan, Paul Revill, Francis Kamwendo, Mohsin Sidat, Honorati Masanja, Helen de Pinho, Edson Araujo

**Affiliations:** 1 School of Nursing, Midwifery & Health Systems, University College Dublin, Dublin, Ireland; 2 School of Medicine, University College Dublin, Dublin, Ireland; 3 Centre for Health Economics, University of York, York, UK; 4 Centre for Reproductive Health, College of Medicine, University of Malawi, Zomba, Malawi; 5 Department of Community Health, Faculty of Medicine, Eduardo Mondlane University, Maputo, Mozambique; 6 Ifakara Health Institute, Dar Es Salaam, Tanzania; 7 Averting Maternal Death and Disability Program (AMDD), Heilbrunn Department of Population and Family Health, Mailman School of Public Health, Columbia University, New York, USA; 8 Health, Nutrition and Population Programme, World Bank, Washington DC, USA

**Keywords:** Human resources, Obstetric care providers, Non-physician clinicians, Job preferences, Malawi, Tanzania, Mozambique, Retention, Discrete choice experiments

## Abstract

**Background:**

Task shifting from established health professionals to mid-level providers (MLPs) (professionals who undergo shorter training in specific procedures) is one key strategy for reducing maternal and neonatal deaths. This has resulted in a growth in cadre types providing obstetric care in low and middle-income countries. Little is known about the relative importance of the different factors in determining motivation and retention amongst these cadres.

**Methods:**

This paper presents findings from large sample (1972 respondents) discrete choice experiments to examine the employment preferences of obstetric care workers across three east African countries.

**Results:**

The strongest predictors of job choice were access to continuing professional development and the presence of functioning human resources management (transparent, accountable and consistent systems for staff support, supervision and appraisal). Consistent with similar works we find pay and allowances significantly positively related to utility, but financial rewards are not as fundamental a factor underlying employment preferences as many may have previously believed. Location (urban vs rural) had the smallest average effect on utility for job choice in all three countries.

**Conclusions:**

These findings are important in the context where efforts to address the human resources crisis have focused primarily on increasing salaries and incentives, as well as providing allowances to work in rural areas.

## Background

The role of the health workforce as a critical pillar of a health system’s ability to meet population healthcare needs has become a major focus of attention, particularly in low and middle-income countries. Until relatively recently human resources for health (HRH) represented a neglected area for research and investment in health systems development [18]. It is only really since the World Health Report 2006 [48], which was devoted almost exclusively to assessing a stated crisis in the global health workforce and the examination of measures to tackle it, that HRH has received greater attention. Such efforts have been motivated by increasingly clear evidence of the relationship between the number and quality of human resources for health and improved health outcomes [2, 42].

The World Health Organization (WHO) identified 57 countries, 36 of which are in sub-Saharan Africa, that fall below the threshold in workforce density required for significant coverage of essential interventions, including those necessary to meet the health-related Millennium Development Goals (MDGs). In most of Africa, there are fewer than five doctors for every 100,000 people, and each year 20,000 health professionals leave their posts to pursue jobs in urban areas, outside of the public health system or outside of their own countries. Africa requires an estimated 140% increase in the total number of doctors, nurses and midwives to ensure adequate coverage of essential health interventions – a shortage of almost one million health workers.

Density of human resources in the health sector, particularly doctors, nurses and midwives, own has been shown to correlate with variation in infant, under-five and maternal mortality rates across countries. Given the current inadequate and uneven distribution of the global health workforce, meeting the MDGs for health, particularly MDG 5 (to improve maternal health) and MDG 4 (to reduce child mortality, will be impossible without substantial increases in human resources. While doctors have the skills necessary to provide emergency obstetric care (EmOC), they are in such limited supply and high demand it is neither practical nor economic that they provide all the care required. As such, the use of mid-level providers (MLPs) including non-physician clinicians (NPCs), all clinical health professionals who are not doctors, is one key strategy to providing quality EmOC, thereby reducing maternal and neonatal deaths.

NPCs have been trained and deployed in 25 of 47 sub-Saharan African countries. Initial research is reporting that NPCs expand cost-effective quality services to under-serviced areas and play a critical role as part of a team of health workers providing care [8, 32, 33]. However, these cadres – a valuable resource - are placed in a vulnerable position because so little attention has been paid to their on-going training and career development. In many low-income countries, NPCs provide much of the EmOC [23], but an enabling environment is needed to continue, expand and improve upon that care.

Recent research has contributed to a greater understanding of the factors affecting the motivation, retention and performance of these cadres [29]. In contrast to a commonly held belief, it appears that financial incentives alone are insufficient as a motivator for health workers ([7, 29]). Instead a range of financial, career development and managerial factors seem to be necessary [49].

McAuliffe et al. [30] show that organizational justice – perceived fairness in decisions, procedures and outcomes – correlated particularly strongly with job satisfaction amongst NPCs.

The range of factors likely to lead to the motivation and retention of NPCs and other health workers now appears to be relatively well understood. What is less well known however is the relative importance of the different factors in determining motivation and retention; how these interact, and how they differ across different cadres of health worker and different settings. In this study we aim to explore such factors for health workers providing obstetric care.

### The DCE literature on employment preferences in sub-Saharan Africa

One method commonly used to identify the relative importance of different attributes is the discrete choice experiment (DCE). DCE is a choice technique based on the assumption that any good or service can be described in terms of its characteristics (attributes) and individuals choose goods and services trading among attributes and their levels [39]. Respondents are presented with hypothetical scenarios and asked to make a sequence of choices between alternatives presented to them. DCEs have been widely used in health services research (see [11]: [9]; for comprehensive reviews) and recently a number of studies have been published focusing on health professionals’ job preferences (see Table 1). Due to the acute shortage of HRH in sub-Saharan Africa in particular and the need to implement more efficient policies to motivate and retain staff, there is a relatively rapid growth of interest in the use of DCEs to determine health workers’ job preferences.

**Table 1:** Attributes and attributes levels for HRH DCE applications

Authors	Country and Sample	Attributes	Attribute levels
Mangham and Hanson [27]	Malawi; 107 registered nurses	Place of work	City, District town
		Net monthly payment	K30.000, K40.000, K50.000
		Availability of material resources	Usually inadequate supply, Usually inadequate supply
		Typical daily workload	Light, Medium, Heavy
		Provision of government housing	No gov. housing provided, Basic gov. housing provided, Superior gov. housing provided
		Opportunity to upgrade qualifications	After 3 years, After 5 years
Hanson and Jack [13]	Ethiopia; 219 doctors and 642 nurses	Geographical location (place of work)	For doctors: Addis Ababa, Zonal capital. For nurses: City, Rural area
		Net monthly pay	Base is salary at average civil service grade, Others multiples of this.
		Government provided housing	None, Basic, Superior
		Availability of equipment and drugs	Inadequate, Improved
		Time commitment following training	1 year, 2 years
		Permission to hold a second job in the private sector (doctors only)	Permitted, Not permitted
		Level of supervision (nurses only)	High, Low
Blaauw et al. [5] Labelled design; presented alternatives described as ‘urban job’ and ‘rural job’	Kenya, S Africa, Thailand; 300 graduating nurses per country	Facility	Urban, Rural
		Salary	Urban – entry salary; Rural – entry salary +10, +20 and +30%
		Training (years of service before study leave)	Varied by country. (e.g. Kenya: No study leave; 1 years study leave after 4 years service)
		Housing	Urban – none, basic; Rural – basic, superior
		Promotion (years of service before promotion)	Varied by country Kenya: 2 years; 4 years S Africa and Thailand: 1 year; 2 years
		Additional benefit	Varied by country. Kenya: Short-term; Permanent contract S Africa: None; Car allowance Thailand: Basic, expanded insurance cover
		Workplace culture	Hierarchical, Relational
Kruk et al. [20]	Ghana; 302 fourth year medical students	Salary	Basic; +30; +50%; Twice basic
		Children’s education	No allowance; Allowance
		Infrastructure, equipment, supplies	Basic; Advanced
		Management style	Unsupportive; Supportive
		Years of work before study leave	Study leave after 5 years of service; After 2 years
		Housing	None; Basic; Superior
		Transportation	Utility car not provided; Provided
Kolstad [19]	Tanzania; 320 clinical officer final year students	Salary and allowances	
		Education opportunities	None; Education opportunity offered after 2; 4; and 6 years
		Location	Dar-es-Salaam; Regional HQ; District HQ; .3 h drive from district HQ
		Availability of equipment and drugs	Sufficient; Insufficient
		Workload	Normal; Heavy
		Housing	None; Decent house provided
		Infrastructure	No utilities; Utilities and mobile coverage
Ageyi-Baffour et al. [1]	Ghana: 298 third-year midwifery students	Salary	Base, base plus 30%
		Children’s education	No allowance, allowance
		Infrastructure, equipment & supplies	Basic, advanced
		Management style	Not supportive, supportive
		Minimum years of work before study leave	2, 5 years
		Housing	Free basic, free superior
		Transportation	No car loan, car loan
Rockers et al., [37]	Uganda: 246 medical students, 132 nursing students, 50 pharmacy students 57 laboratory students	Salary	4 levels customised for each cadre
		Facility Quality	Basic, advanced
		Housing	No housing, free basic housing, housing allowance
		Length of commitment	2, 5 years
		Support from manager	Not supportive, supportive
		Future tuition	No provision, full tuition fees
Bocoum et al., [6]	Burkina Faso: 315 regional health workers	Regionalised Recruitment strategy	Continue, cancel, commit 5, 10 years
		Motivation allowance	3 levels from €33.6-€64.1
		Medical coverage	75% reduction for lab exams. 80% reduction lab and medicines; free medciation and lab exams
		Work equipment	Sufficient quality equipment, insufficient, sufficient quantity but poor quality
		Housing	Free housing, no housing, 25% increase in housing allowance
Robyn et al. 2015 [36]	Cameroon: 351 medical students, nursing students and health workers	Accessability/connectivity to the city	Poor; good
		Health Facility infrastructure	Lack of; adequate
		Lodging	None; good quality housing
		Career development	No prefential access to ongoing training; preferential access
		Salary	Base; base + 255; base +50%; Base + 75%
		Job assignment in an urban area	Uncertain; automatic after 3 years
Honda & Vio [17]	Mozambique: 334 non-physician clinicians, 123 students	Place of work	Rural, Capital city; provincial city
		Monthly salary	Base salary, base plus 50%; base plus 100%
		Housing	None; Government housing
		Loan for housing or land	Not available; available
		Formal Education	None offered; offered after 5 years only
		Skills development	No in-service training; regular in-service training
		Availability of equipment & Medicine	Inadequate;adequate
		Private practice	Part-time allowed; allowed outside hours
Takemura et al. [44]	Kenya: 57 clinical officers	Quality of the Facility	Basic; Advanced
		Education opportunities	1 year study leave after 2 years; after 5 years
		Housing allowance	Insufficent to afford basic; sufficient for superior
		Monthly basic salary	10% additional; 30% additional
		Promotion eligibility	In 2 years; in 3 years

Table 1 summarizes the major works in this area. One of the earliest uses of DCEs to investigate the job preferences of health workers in Africa was Mangham and Hanson [28]. This work was undertaken in the context of a major Government drive to increase the salaries of health workers. The results of the discrete choice experiment found that there were relatively few nurses whose preferences were dominated by a single attribute, and all six attributes had a statistically significant influence on the nurses’ preferences. The nurses were willing to trade between job attributes, and therefore willing to forego pay increases to obtain improvements in their non-monetary benefits or working conditions. The opportunity to upgrade qualifications, provision of basic government housing (compared with none) and increases in net monthly pay had the greatest impact on the utility associated with a particular job.

A similar DCE was conducted in Ethiopia [13], which was particularly focused on identifying factors affecting labour supply of doctors and nurses in rural areas. For doctors, they found that higher wages and quality housing incentives had the biggest impact on their willingness to work in rural areas. For nurses, availability of medical equipment and supplies were more likely to attract them to rural areas. Interestingly, they also found that married doctors valued a job in Addis Ababa three times as highly as their single counterparts, whereas younger doctors placed a higher value on reduced time spent working in remote rural areas to meet training payback commitments than their older colleagues.

A multicountry DCE study in Kenya, South Africa and Thailand also examines the effectiveness of different job attributes in attracting graduating nurses to work in rural areas [5]. A labelled design was used, with the two job alternatives presented being described as a ‘rural job’ and an ‘urban job’, so attributes differed across presented alternatives. In Kenya and South Africa training opportunities and rural allowances were shown to be particularly important, whereas in Thailand health insurance coverage was estimated to have the greatest impact. Only a minor preference for relational over hierarchical work cultures was reported (odds ratio of 1.2 for choosing the job in Kenya and South Africa, and 2.0 in Thailand). Given the variations in preferences according to age and marital status found in Hanson & Jack’s study, caution should be employed in generalising findings from studies with newly graduating health professionals to the health workforce already employed in the service. An attribute proving attractive to a newly graduated health professional may not have the same potential to retain an experienced, possibly demotivated health worker in the system.

DCEs have also been employed to estimate job preferences, albeit amongst students, in West Africa. Kruk and her colleagues examine the factors that affect preferences of medical students for rural postings in Ghana [20]. The strongest predictors of job choice were improved infrastructure, equipment and supplies; supportive management; and the provision of housing. The choices of women were shown to be particularly influenced by supportive management style whereas for men superior housing was considered more important. Kruk et al. interpret the student’s valuing of non-monetary attributes over high remuneration as a social desirability effect of the study. The paper also suggests that the students’ interpretation of ‘management style’ is not clear and may indicate concerns about being ‘forgotten’ in rural areas when it comes to promotion and training opportunities. Rockers et al. [37] study of students in Uganda found choice of job posting was strongly influenced by salary, facility quality and manager support, relative to other attributes and they conclude that salary is not the only important factor health workers consider when deciding where to work. However, Robyn et al. [36] found that among medical and nursing students a rural retention bonus of 75% of base salary and improved health facility infrastructure respectively were the attributes with the largest effect sizes. Among medical doctors and nurse aides, a rural retention bonus of 75% of base salary was the attribute with the largest effect size. On the other hand, improved health facility infrastructure, was the attribute with the largest effect size among the state registered nurses surveyed. Ageyi-Baffour et al. [1] in a study of midwifery students identified: 1) study leave after 2 years of rural service; 2) an advanced work environment with reliable electricity, appropriate technology and a constant drug supply; and 3) superior housing (2 bedroom, 1 bathroom, kitchen, living room, not shared) as the top three motivating factors to accept a rural posting in Ghana.

All the initial uses of DCEs to examine employment preferences in the African context focused on traditional cadres of health worker (doctors and nurses) or students. The work of Kolstad [19] examines preferences of NPCS (clinical officers) in Tanzania. As with several of the aforementioned studies, the particular policy interest was attracting health workers to rural areas. HR management was not included as a job attribute, but the study found wages (including hardship allowances) and opportunities for continued education to be particularly strong predictors of choice. Similarly a study with clinical officers in Kenya [44] found that educational opportunities i.e. a 1-year guaranteed study leave after 3 years of service would have the greatest impact on retention, followed by good quality health facility infrastructure and equipment and a 30% salary increase. A larger study in Mozambique [17] included 334 non-physician clinicians (trained for 3 or 5 years) and 123 student cohorts of the same cadres. The study drew from the design of the study reported in this paper and therefore included a broader range of attributes than previous studies (see Table 1). Their results indicated that the provision of basic government housing had the greatest impact on the probability of choosing a job at a public health facility, followed by the provision of formal education opportunities and the availability of equipment and medicine.in the facility. Housing also featured strongly in a study of 315 regional health workers in a Burkina Faso study [6].

Although the existing literature is based on experiments across countries and on different health worker cadres, some common findings are emerging. Opportunities for education and professional upgrading appear to be a leading determinant of choice; location is often crucial; and pay is also important but is not as strong a predicator as many may have previously believed. Human resource management (HRM) does not feature prominently within experiments to date; and although some papers have found management style is a strong predictor of choice [20], others have found less of an effect [5, 13].

Mandeville et al. [26] in a recent systematic review of the use of DCEs to inform health workforce policy called for more studies that focus on a wider range of health workers. In many countries e.g. Tanzania, Malawi, Ethiopia, Mozambique non-physician clinicians (e.g. clinical officers, surgical technicians) and mid-level cadres comprise the majority of the workforce. Our study includes all cadres engaged in the provision of EMOC in public facilities across 3 countries and therefore adds to our understanding of what motivates these different cadres of health workers.

This paper presents findings from large sample discrete choice experiments to examine the employment preferences of all cadres providing obstetric care in the public health systems in Malawi, Mozambique and Tanzania. The study is one component of the Health System Strengthening for Equity Study (HSSE). HSSE used a systems approach to explore how NPCs function within the delivery system for EmOC. Drawing on the WHO framework for monitoring health systems, HSSE focused on addressing the six building blocks necessary for a functioning health system – generating the evidence that explored the gaps and constraints in the system and using this information to advocate for evidence based policy changes at global, regional and national levels. The DCE component of the study was aimed at identifying the importance of different factors in the work environment that are considered influential in the motivation and retention of staff.

This study addresses the gaps in the extant literature by focusing on cadres that are currently providing the majority of healthcare and by including potentially important motivators such as human resources management and professional development. In addition the study comprises a large sample across three countries, where the majority of previous studies (apart from Blaauw et al.’s [5] study across three countries that focused only on graduating nurses) are single country studies.

## Methods

### Ethics statement

The study was approved by the Institutional Review Board of Columbia University, New York; Global Health Ethics Committee Trinity College, Dublin; and the Institutional review boards of College of Medicine, Malawi, Eduardo Mondlane University, Mozambique and Ifakara Health Institute, Tanzania.

### Discrete choice experiments

This paper explores health workers’ preferences for job attributes using a discrete choice experiment (DCE). The DCE method has its foundations in probabilistic choice and random utility theory [12]. It enables observation of individuals’ preferences in situations where either the market does not exist (e.g. for new goods and services), is imperfect (e.g. public goods, such as parks); or when there is insufficient variation between attributes to permit accurate estimation of demand functions (as is the case for employment preferences). It is assumed that when faced with alternatives an individual will choose that which yields the greatest utility. The true utility an individual derives from an alternative is not directly observable, but is assumed to be composed of utility associated with constituent attributes that can be observed [21]. The individual is assumed to be rational and consistent in his/her choices.

In human resources applications, DCEs are used to describe hypothetical job alternatives (or choice scenarios) presented to respondents who are requested to choose one. Each respondent evaluates a series of choice scenarios carefully designed in order to have some desirable statistical properties [24]. The multiple choices made by each respondent permit measurement of the relative importance of the job attributes upon which health workers make their choices. DCEs, therefore, provide valuable evidence to inform policies to attract and retain human resources for health since they enable observation of what influences health workers’ employment decisions.

### DCE experimental design

The design of DCEs involves different stages, from the selection of attributes and attribute levels to the construction of choice scenarios [43]. The aim is to construct hypothetical scenarios that are meaningful and important to the respondents, without resulting in heavy cognitive burden, whilst at the same time being statistically efficient [4, 25].

The first step in the process is the selection of a valid and comprehensive set of attributes and attribute levels related to the choices being analysed. All possible combinations of attribute and levels are enabled through a factorial design, and a fraction of possible combinations are selected to be included in the choice surveys (this is known as a fractional factorial design). The literature on experimental design for DCE is large and continuously evolving; with contributions coming from diverse fields such as environmental economics, marketing, and transportation economics [24, 47].

The selection of attributes for this study was based on previous research (qualitative interviews) conducted with mid-level cadres in Malawi [30] which showed that how people were treated by their managers, their involvement in decision making and opportunities for development and advancement (all elements of human resource management and professional development) were amongst the strongest predictors of job satisfaction. A strong correlation between management support and intention to leave the job [29] suggested that good human resources management might be an important consideration in job choice. Housing, pay, urban location and availability of resources and equipment required for the job were the most commonly reported attributes of importance in previous studies conducted with similar populations (e.g. [13, 28]). Table 2 below presents the set of attributes and their respective levels; a detailed description of each attribute is presented in Appendix 1. Also presented is the variable coding scheme used for statistical analysis, discussed below in the section on model fitting.

**Table 2:** Attributes and attribute levels for job alternatives – three countries

Attribute	Possible levels	Variables for analysis	Variable coding
Location	*Urban* *Rural*	location	*0* = *rural* *1* = *urban*
Net monthly pay	*Base* *1.5* × *base* *2* × *base*	pay1	*0* = *base salary* *1* = *1.5* × *base salary or 2* × *base salary*
		pay2	*0* = *base salary or 1.5 v base salary* *1* = *2* × *base salary*
Housing	*None* *Basic* *Superior*	house1	*0* = *no housing* *1* = *basic or superior housing*
		house2	*0* = *no housing or basic housing* *1* = *superior housing*
Equipment and Drugs	*Inadequate* *Improved*	equip	*0* = *Inadequate* *1* = *Improved*
Continuing Professional Development	*Limited* *Improved*	PD	*0* = *Limited* *1* = *Improved*
Human Resources Management	*Poor* *Functioning*	HRM	*0* = *Poor* *1* = *Functioning*

All possible combinations of attributes and attribute levels (i.e. a full factorial design) would result in 144 possible scenarios or job descriptions (2^4^ × 3^2^, i.e. four attributes with two levels and two attributes with three levels). In order to have a manageable number of scenarios, a fractional rather than full factorial design was used. A set of choices was selected to allow the main effects (the effect of each independent variable on the dependant variable) to be explored. A constant comparator method was used i.e. holding one job specification constant while changing the levels of the attributes in the second job specification. In total 15 choice sets were presented. In DCE applications in the health research arena there has been a move towards the use of optimal designs and the use of SPEED software to generate orthogonal designs. A recent review of DCE designs [3] identified fractional factorial designs as the most commonly used for DCE applications. In addition they found the mean number of attributes to be 5 and the mean number of choice sets to be 14. We did not include an opt-out option in the design. The rationale for employing a forced choice is that although an opt-out option can reduce biases in parameter estimates, it cannot provide sufficient information on respondents’ preferences for the attributes if too many respondents choose the opt-out option [40].

Field staff received a one-week training on all steps in the data collection process. This included a trip to the field to pilot test the instrument on a small sample of health workers. Although the survey was designed to be self-administrated, field staff were required to remain in the facility during the data collection period to explain the contents of the survey and answer any questions that staff might have. Ensuring a common understanding of the attributes and levels and the provision of standard explanations across all sites was emphasised to fieldworkers during training. The descriptions of attributes and attribute levels are contained in Appendix 1. This was included in the survey instrument and respondents were instructed to read and make sure they understood these before completing the questionnaire.

### Sample

The primary target for the DCE was health care workers who had performed at least one of the EmOC signal functions in the previous three months; thus the focus was on maternity staff, as well as health care workers who provide surgical services, such as caesarean section. Since it was not possible to randomly sample healthcare workers themselves, guided by existing staffing levels, the project randomly sampled hospitals and health centres to be visited to approach the minimum target of 500 health care workers per country for the provider survey. Hospitals were intentionally oversampled because the majority of EmOC is provided in hospitals rather than health centres. In Malawi, a near-national sample of facilities (*N* = 84) intended to provide EmOC services was identified and included central, district, rural and CHAM (faith-based organisations) -operated hospitals and a randomly sampled urban and recently upgraded health centre designated to provide EmOC. A few districts/facilities were excluded in Malawi due to their recent participation in another human resources study in which similar data had been collected from health workers. In Tanzania, due to the size of the country, cluster sampling was employed. One region was randomly selected in each of the eight geographic zones and all districts within those eight regions were then included in the sampling frame. The primary hospital serving the district was identified for inclusion; either the government-run district hospital or voluntary agency-run (VA) designated district hospital (DDH). In some districts that also contain the regional headquarters, the regional hospital was included in the sample when there was no district hospital serving the community. One health centre (HC) was randomly selected in each district, thus there were two facilities from each district in the study (*N* = 90). In Mozambique, a near national sample of general, district and rural hospitals was included to maximise the potential participation of the NPC cadre tecnico de cirurgia. In addition, two to three health centres (type 1 and type 2) providing maternity care, and therefore at least some basic EmOC functions, were randomly selected in each district for inclusion in the study (*N* = 138).

### Data collection

Data collection was conducted in the three countries during 2008–09. In each of the selected facilities staff were deemed eligible to participate if they were present during the study visit and reported having provided at least 1 of the 9 EmOC signal functions within the previous 3 months, and had granted informed consent. There are 7 signal functions for basic EmOC (parenteral antibiotics, parenteral utertonics, parenteral anti-hypertensives, removal of retained products, manual removal of placenta, assisted vaginal delivery, neonatal resuscitation), and 9 signal functions for comprehensive EmOC (the basic 7, plus caesarean delivery and blood transfusion).

**Fig. 1:**
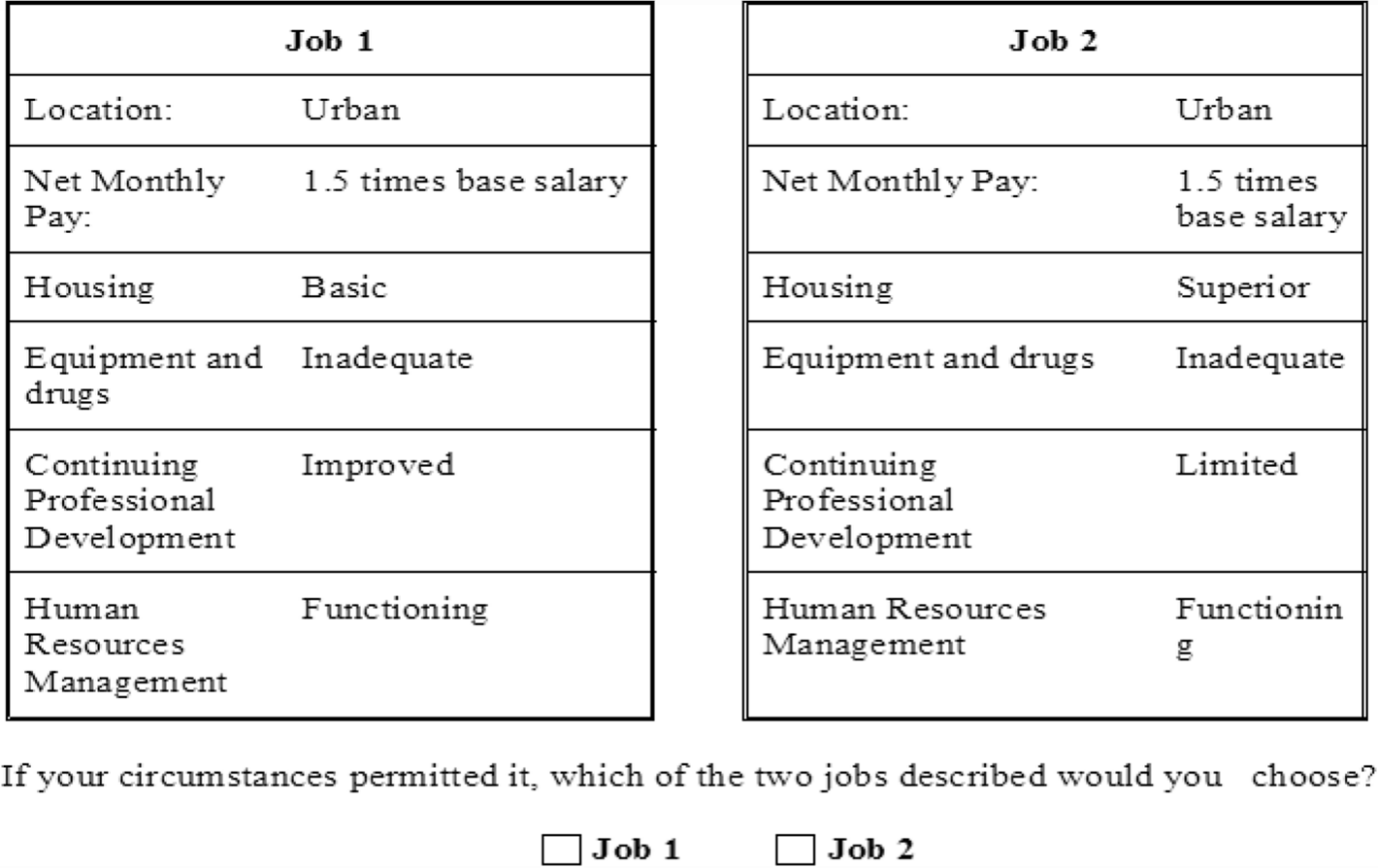
Example of a discrete choice experiment question (choice set)

The questionnaire was self-adminstered in the English language. Details of the data collection procedure are provided in Appendix 2. Each respondent was asked to evaluate 15 choice sets and chose one job description; each choice set containing two job descriptions (see Fig. 1 contains an example of choice set). Besides the choice experiment the questionnaire also included demographic data.

### The mixed logit model

Discrete choice models are Random Utility Models (RUMs) that are widely used for the analysis of discrete choice experiments. Three underlying assumptions of discrete choice models are that (i) choice is discrete (individuals either choose a particular alternative or not), (ii) the utility for an alternative is a random variable that varies over individuals and (iii) in a choice situation, individuals choose the alternative for which their utility is maximized.

The aim of discrete choice models is to estimate the probability of an individual choosing one alternative over the other alternatives presented in the choice scenario [15, 25]. Individuals choose goods and services that yield the highest utility (or satisfaction). Therefore, the choice between alternatives in a choice experiment is based on the combination of attributes and attribute levels that results in an increase in utility for the respondents ([27, 38]). The task is then to estimate parameters that determine the relative importance of different attributes affecting the choice process.

Conditional (or multinomial) logit models are discrete choice models that have been utilized in many fields of research, from marketing to medicine. In recent times however, these models have been superseded somewhat by the more flexible mixed logit model. The mixed logit model has become popular following the development of simulation methods that enable it to be estimated more readily, and following the integration of these methods into popular software tools [14]. The mixed logit is a highly flexible discrete choice model that can approximate any random utility model [31]. Hensher and Greene [14] and Train [46] describe this model in detail. A more detailed description of the model and its parameter estimation is contained in Appendix 3.

### Model fitting

Mixed logit models were fitted to the discrete choice data from each country to estimate job preferences. All choice scenarios presented to individuals contained two unlabelled alternatives (two job descriptions). Each job was described by six attributes, four of which had two levels (location, equipment, professional development and HRM) and two of which had three levels (pay and housing).

All job attributes were represented as categorical measures (Table 2) and therefore were coded as dummy variables for statistical analysis. Attributes were coded for analysis as binary (dummy) variables. Each two-level attribute was represented by a single binary variable, while each three-level attribute (pay and housing) was represented by two binary variables. Table 2 shows the attribute coding system used in the analysis. Pay was included as a categorical, rather than a continuous predictor, to allow for the possibility of a non-linear effect of pay on utility. It was considered likely that the added utility of *1.5* × *base* over *base* pay, was not the same as the added utility of *2* × *base* over *1.5* × *base*.

Binary mixed logit models were fitted to estimate the probability of an individual choosing a given alternative (job 2) over the other (job 1). Normally distributed random coefficients were specified for each of the eight attribute variables.

It is possible that an individual’s utility for particular job attributes may differ depending on observed characteristics of that individual. For example, it could be possible that older individuals place a higher value on superior quality of housing, or that females have a stronger preference for jobs with improved availability of continuing professional development. To allow effects such as these to be captured, we tested for fixed effect interactions between each alternative-specific attribute (Table 2) and each of the individual-specific characteristics listed in Table 3.

**Table 3:** Sample demographics for each country

		Malawi (*N* = 602)	Mozambique (*n* = 569)	Tanzania (*N* = 801)
Frequency (and percentage)	current location			
	rural*	276 (45.85%)	569 (100%)	637 (79.53%)
	urban	326 (54.15%)	0 (0%)	164 (20.47%)
	facility			
	health center*	65 (10.8%)	378 (66.43%)	257 (32.08%)
	hospital	537 (89.2%)	190 (33.39%)	544 (67.92%)
	missing	0 (0%)	1 (0.18%)	0 (0%)
	gender			
	male*	203 (33.72%)	103 (18.1%)	202 (25.22%)
	female	398 (66.11%)	463 (81.37%)	589 (73.53%)
	missing	1 (0.17%)	3 (0.53%)	10 (1.25%)
	cadre			
	basic	0 (0%)	149 (26.19%)	165 (20.6%)
	mid*	380 (63.12%)	331 (58.17%)	292 (36.45%)
	high	215 (35.71%)	79 (13.88%)	342 (42.7%)
	missing	7 (1.16%)	10 (1.76%)	2 (0.25%)
Summary	age			
	min	21	20	20
	mean	34.13	32.46	39.75
	max	73	60	63
	missing	33	24	47

*baseline category

Note that the first four individual-specific characteristics in Table 3 are categorical, while the fifth is a numeric variable. The baseline category for each categorical variable is marked in the table (*) and the variable is therefore represented by the inclusion of dummy variables for the other categories.

Health workers were grouped into basic, mid and high level cadres within each country, as defined in Table 4. Note that health workers in Malawi were grouped into mid and high level cadres only and therefore only a single dummy variable was required for cadre (the baseline category is mid-level cadre, while a dummy was included for high level cadre).

**Table 4:** Grouping of cadres for statistical analysis

		Tanzania	Malawi	Mozambique
Cadre group	High	Registered nurse Registered nurse midwife Registered public health nurse Clinical Officer Assistant Medical Officer General Doctor Doctor Specialist	Registered nurse Registered nurse midwife Clinical Officer Medical Assistant General Doctor Doctor Specialist	Nurse (higher degree) General Doctor
	Mid	Enrolled Nurse Enrolled Nurse Midwife Enrolled public health nurse	Enrolled Nurse Enrolled Nurse Midwife Nurse Midwife Technician	Mid-level nurse Mid-level MCH nurse Nurse midwife Basic level nurse Basic level MCH nurse
	Basic	MCH Aide Medical Attendant Nursing Assistant		Elementary level nurse Elementary midwife Medical Agent

Fitting a mixed logit model with eight random coefficients is highly computationally intensive. It was therefore infeasible here to perform variable selection on all fixed effect interaction terms under the specified mixed logit model. Instead, bootstrap variable selection was carried out using conditional logit models (assuming that all coefficients were fixed). For each of the three country datasets, 200 bootstrap samples were drawn from the data and a forward greedy search algorithm was carried out to select the fixed effect interaction terms that should be included. The Bayesian Information Criterion (BIC) proposed by Schwarz [41] was used to decide whether covariates should be added or removed from the model. For each bootstrap sample, the greedy search algorithm proceeded as follows:

1. Define the set of candidate variables as the interaction of each alternative specific attribute with each relevant individual-specific characteristic.
2. Define the initial model to be the conditional logit model fitted including all alternative-specific attributes and excluding all candidate variables (interactions).
3. Calculate the change in BIC that would occur by adding each candidate variable to the initial model. Add the candidate variable to the model that gives the largest increase in BIC.
4. Repeat step 3. At this stage there should be two candidates in the current model.
5. Propose to remove a candidate variable from the model. If removing any of the candidate variables from the model increases the BIC, then remove the candidate variable that gives the largest increase in BIC. Otherwise, don’t remove a candidate.
6. Propose to add a candidate variable to the model. If adding any of the candidate variables to the model increases the BIC, then add the candidate variable that gives the largest increase in BIC. Otherwise, don’t add a candidate.
7. Repeat steps 5 and 6 until no further changes are made to the model. The candidates included in the model at this stage are selected for inclusion.

A similar variable selection strategy to the above was used in Raftery and Dean [34] and in Galligan et al. [10] to select variables for inclusion in clustering and classification models respectively. Results across the 200 bootstrap samples were compiled. Fixed effect interaction terms that were chosen in 50% or more of the bootstrap samples were considered to be important, and hence were selected for inclusion in the mixed logit model for that country.

Mixed logit models were fitted with varying numbers of Halton draws [45], starting at 500 draws and increasing the number of draws by 500 until convergence of the parameter estimates was reached. A large number of draws was required for each dataset, attributable to the eight random coefficients for which distributional parameters must be estimated.

Likelihood ratio tests were carried out to test for the inclusion of correlated (vs. independent) random effects. In all cases, likelihood ratio tests provided evidence that correlated random coefficients improved model fit (Table 5) and therefore correlated random coefficients have been included in all mixed logit models presented below.

**Table 5:** Likelihood ratio tests comparing models fitted with uncorrelated, and correlated, random coefficients

Country model	Log likelihood (uncorrelated random coefficients)	Log likelihood (correlated random coefficients)	Likelihood ratio test
Malawi	−3524.4	−3439.2	*X* ^2^ = 170.4, df = 28, *p* < 0.001
Mozambique	−3899	−3828.8	*X* ^2^ = 140.51, df = 28, *p* < 0.001
Tanzania	−5642.7	−5508.4	*X* ^2^ = 268.45, df = 28, *p* < 0.001

### Software

Conditional logit models were fitted in the *mlogit* package in R (R: A language and environment for statistical computing). Mixed logit models were fitted here using the *mixlogit* command [16] in Stata Version 12.1.

## Results

### Malawi

A total of 602 health workers (response rate 87%) in Malawi completed the discrete choice experiment. Thirty-four health workers were missing information on the individual-specific variables included in the selected mixed logit model, so this model was fitted using data on 568 individuals. Most of these individuals responded to all 15 choice situations, but 13 individuals (2.3%) were missing one or more responses.

The fixed effect interaction terms selected most frequently from 200 bootstrap samples were: the interaction between gender and HRM, the interaction between age and PD, and the interaction between the individual’s current job location (rural vs urban) and the location of the jobs they were choosing between. These interaction effects were selected in 82, 68.5 and 55% of bootstrap samples respectively. Other interaction effects were selected in less than 50% of samples and thus were omitted from the mixed logit model.

Mixed logit parameter estimates converged at 3000 Halton draws. Table 6 shows the estimated coefficients with 95% confidence intervals, Z statistics and corresponding *p*-values.

**Table 6:** Mixed logit model results for DCE in Malawi

Coefficient	Estimate (95% confidence interval)	Z	*p*-value
Fixed			
gender*HRM	0.537 (0.059, 1.015)	2.2	0.028
age*PD	−0.03 (−0.05, −0.01)	−2.99	0.003
current_location* location	0.506 (0.184, 0.829)	3.08	0.002
Random (Mean)			
location	−0.653 (−0.927, −0.378)	−4.66	<0.001
pay1	2.39 (2.056, 2.723)	14.03	<0.001
pay2	1.78 (1.318, 2.242)	7.55	<0.001
house1	2.507 (2.108, 2.906)	12.31	<0.001
house2	0.67 (0.336, 1.004)	3.93	<0.001
equip	2.184 (1.844, 2.524)	12.59	<0.001
PD	3.851 (3.058, 4.645)	9.51	<0.001
HRM	3.26 (2.662, 3.857)	10.69	<0.001
Random (Standard deviation)			
location	0.592 (0.242, 0.943)	3.31	0.001
pay1	1.288 (0.955, 1.62)	7.59	<0.001
pay2	2.276 (1.825, 2.726)	9.9	<0.001
house1	1.456 (1.05, 1.862)	7.03	<0.001
house2	1.767 (1.396, 2.139)	9.32	<0.001
equip	1.74 (1.441, 2.038)	11.43	<0.001
PD	2.079 (1.706, 2.453)	10.91	<0.001
HRM	2.09 (1.687, 2.493)	10.17	<0.001

As one might expect, health workers showed strong preferences for jobs with a better than basic salary. The estimated effect on utility of a job with a salary of twice the base level compared with a job paying the base salary is 4.1 (the sum of the coefficients for *pay1* and *pay2*), making this job attribute the one which health workers found most attractive overall. The estimated standard deviation for *pay1* and *pay2* are 1.3 and 2.3 respectively, indicating that there was a much larger variability in health worker preferences when it came to jobs with a salary of twice the basic level than there was in preferences towards jobs with a salary of 1.5 times the base level. Based on the Normal distributions fitted to random coefficients (means and standard deviations shown in Table 6), an estimated 97% of health workers prefer jobs with a salary of 1.5 times the base salary, while a lower percentage of 78% are estimated to prefer a job with a salary of twice the base level (over a salary of 1.5 times the base level). Although salary was valued most highly by health workers, other job attributes appear to be almost equally important to workers.

The mean coefficient for *HRM* is 3.3 (estimated for males), highlighting the strong preference of health workers on average for jobs where there is a functioning system of human resource management. Females prioritize HRM even more strongly than males, with an estimated mean coefficient of 3.8 (sum of coefficients for *HRM* and gender*HRM). However, there is also a large variability in the preferences of health workers for this attribute (estimated standard deviation = 2.09).

Strong preferences for HRM were closely followed by preferences for the availability of continuing professional development (*PD*), which varied with age. For the youngest person in the sample (aged 21), the estimated mean coefficient for professional development is 3.2, while the estimated mean coefficient for the oldest person in the sample (aged 73) is just 1.7. This indicates that while improved (as opposed to limited) availability of continuing professional development was one of the most valued job attributes for health workers in Malawi, this was significantly more important for younger health workers than for older workers.

The coefficient mean for *house1* is the next largest, indicating that housing had a strong influence on health workers’ job choices, on average. An estimated 96% of health workers preferred jobs with some form of housing provided. The large positive coefficient for house1 (2.507) and the much smaller coefficient for house2 (0.67) indicate that, although health workers had strong preferences for jobs that provided housing (either basic or superior) compared with jobs that provided no housing, the standard of housing provided (basic vs. superior) were not so important to health workers on average.

Health worker preferences were divided when it came to job location (rural vs. urban), and preferences differed for health workers currently based in rural and urban health facilities. Based on the negative coefficient mean (−0.653) for location and coefficient standard deviation (0.592), it’s estimated that approximately 86% of health workers located in rural health facilities also preferred jobs in rural facilities. For health workers in urban facilities, it’s estimated that 60% preferred jobs in a rural location. Despite these differences however, the estimated coefficient means and standard deviation are relatively small, indicating that location was the least important job attribute to health workers.

### Mozambique

A total of 569 health workers (response rate 97%) in Mozambique participated in the discrete choice experiment. Only one individual-specific variable (*basic*) was selected for inclusion in the final model, for which ten of the 569 health workers had missing values. Therefore, the selected mixed logit model was fitted using choice data on 559 health workers. Most of these individuals responded to all 15 choice situations, but 26 individuals (4.7%) were missing one or more responses.

Fixed effect interactions were included in the final model between *basic* (basic level cadre) and *equip*, and between *basic* and *PD*. Interactions with the indicator for high level cadres was not selected for inclusion in the final model. Therefore, the basic*equip and basic*PD interaction terms contrast the preferences of basic level cadres with those of mid and higher level cadres for jobs with improved availability of equipment and drugs, and with improved availability of continuing professional development.

Mixed logit parameter estimates converged at 3000 Halton draws. Table 7 shows the estimated coefficients with 95% confidence intervals, Z statistics and corresponding *p*-values.

**Table 7:** Mixed logit model results for DCE in Mozambique

Coefficient	Estimate (95% confidence interval)	Z	*p*-value
Fixed			
basic*equip	−0.703 (−1.097, −0.309)	−3.5	<0.001
basic*PD	−0.607 (−1.019, −0.194)	−2.88	0.004
Random (mean)			
location	0.056 (−0.148, 0.261)	0.54	0.589
pay1	1.097 (0.887, 1.306)	10.24	<0.001
pay2	0.582 (0.191, 0.973)	2.92	0.004
house1	1.505 (1.199, 1.81)	9.64	<0.001
house2	0.069 (−0.188, 0.326)	0.53	0.599
equip	1.9 (1.616, 2.184)	13.12	<0.001
PD	2.305 (2.015, 2.595)	15.6	<0.001
HRM	1.979 (1.598, 2.36)	10.19	<0.001
Random (standard deviation)			
location	0.485 (0.149, 0.822)	2.83	0.005
pay1	1.055 (0.798, 1.312)	8.05	<0.001
pay2	1.829 (1.444, 2.214)	9.32	<0.001
house1	1.55 (1.197, 1.903)	8.61	<0.001
house2	1.14 (0.81, 1.471)	6.76	<0.001
equip	1.434 (1.18, 1.688)	11.07	<0.001
PD	1.433 (1.139, 1.728)	9.53	<0.001
HRM	1.615 (1.269, 1.961)	9.16	<0.001

Improved (as opposed to limited) opportunities for continuing professional development is estimated to be the most important job attribute on average for mid and high level cadres in Mozambique, with an estimated mean coefficient of 2.3 for *PD*. For basic level cadres, this job attribute was still important on average but less so, with a mean coefficient estimate of 1.7 (=2.3–0.6).

As well as opportunities for professional development, health workers (basic, mid and high level cadres) showed strong preferences on average for jobs described as having a functioning system of human resource management, with an estimated coefficient mean of approx. 2.0 for this job attribute. Mid and high level health workers showed similarly large preferences for jobs that were described as having improved availability of equipment and drugs (coefficient mean = 2). In contrast, basic level cadres valued this job attribute less (coefficient mean = 1.2) than they did other attributes such as provision of housing.

A job with housing provided was more attractive to health workers on average than a job with a salary that was 1.5 times the basic level, indicated by the coefficient mean of 1.5 for *house1* and 1.1 for *pay1*. Superior (compared with basic) quality housing was not valued highly by health workers on average, with an estimated coefficient mean of just 0.1 for this job attribute.

The coefficient mean of 0.6 estimated for *pay2* suggests that a job with a salary that was twice the basic level was valued more highly on average than a job with a salary that was 1.5 times the basic level, as one might expect. However, the largest variability in preferences overall was seen for this variable (*pay2*) with an estimated coefficient standard deviation of 1.8. This large variability suggests that while health workers on average showed a moderate preference for this attribute, there are some who valued it it lot more or a lot less than others.

With regard to job location (urban vs. rural), the coefficient distribution is estimated to be centred close to zero with a mean of 0.06, with a standard deviation of 0.5. This appears to be the least influential job attribute of those considered. Based on the fitted random coefficient distribution with mean 0.06 and standard deviation 0.5, an estimated 55% of health workers were estimated to prefer jobs located in an urban setting, while an estimated 45% prefer jobs in a rural location.

### Tanzania

A total of 801 health workers (response rate 93%) in Tanzania participated in the discrete choice experiment. Only two individual-specific variables (*high* and *fc*) were included in the final model, on which two individuals were missing values. Therefore, the selected mixed logit model was fitted using choice data on 799 individuals. Most (95%) of these individuals responded to all 15 choice situations, with just 36 individuals missing one or more responses.

Fixed effect interactions were included in the final model between *fc* (type of facility in which health worker is based – health center vs hospital) and job *location*, and between *pay1* and *high* (indicator for high level cadre). Since the interaction with *basic* was not selected for inclusion in this final model, the high*pay1 interaction term contrasts the preferences of high level cadres with those of basic and mid level cadres for jobs with a salary that is at least (1.5 × base) as opposed to a basic salary.

Mixed logit parameter estimates converged at 3500 Halton draws. Table 8 shows the estimated coefficients with 95% confidence intervals, Z statistics and corresponding *p*-values.

**Table 8:** Mixed logit model results for DCE in Tanzania

Coefficient	Estimate (with 95% confidence interval)	Z	*p*-value
Fixed			
fc*location	0.457 (0.196, 0.718)	3.44	0.001
high_pay1	0.388 (0.122, 0.654)	2.86	0.004
Random (mean)			
location	−0.122 (−0.349, 0.105)	−1.06	0.291
pay1	0.944 (0.4570.731, 1.158)	8.66	<0.001
pay2	0.451 (0.135, 0.766)	2.8	0.005
house1	1.308 (1.087, 1.529)	11.59	<0.001
house2	−0.308 (−0.504, −0.112)	−3.09	0.002
equip	1.478 (1.262, 1.694)	13.41	<0.001
PD	1.453 (1.253, 1.652)	14.27	<0.001
HRM	2.053 (1.736, 2.371)	12.69	<0.001
Random (Standard deviation)			
location	0.8 (0.579, 1.02)	7.09	<0.001
pay1	0.964 (0.692, 1.236)	6.94	<0.001
pay2	1.166 (0.898, 1.435)	8.51	<0.001
house1	1.363 (1.139, 1.587)	11.92	<0.001
house2	1.495 (1.165, 1.825)	8.88	<0.001
equip	1.408 (1.179, 1.637)	12.05	<0.001
PD	1.442 (1.237, 1.648)	13.79	<0.001
HRM	1.913 (1.63, 2.196)	13.26	<0.001

The job attribute most highly valued by health workers on average was a functioning system of human resource management, which had an estimated coefficient mean of 2.1 and standard deviation of 1.9. Therefore, based on the Normal distribution fitted to this random coefficient, it’s estimated that 86% of the health worker population value this job attribute, with some health workers placing a very high value on this attribute.

The average health worker is estimated to place less value on a job with a high salary than a job with improved availability of equipment and drugs and improved opportunities for continuing professional development. For basic and mid level cadres, the effect of a job with a salary of twice the basic level on utility (compared with a job of basic salary) is 1.4, lower than the estimated average effects for *PD* and *equip*, each with a coefficient mean of 1.5. High level cadres, in contrast, are estimated to place a higher value on a job with a better than basic salary, and have a coefficient mean of 1.8 for the effect on utility of a job with twice the basic salary.

With regard to job location, the preferences of health workers in different types of facilities are estimated to differ. Hospital workers appear to have stronger preferences on average for jobs in an urban location than health center workers. Approximately 56% of health facility workers are estimated to have preferences for jobs in a rural location, compared with an estimated 34% of hospital workers. The relatively small coefficient mean and standard deviation for *location* indicates that location was the least important job attribute to health workers.

Health workers placed a relatively high value on jobs with provided housing. An estimated 87% of health workers prefer jobs with housing provided over jobs without provided housing, based on the coefficient distribution fitted for *house1* with mean 1.3 and standard deviation 1.36 (Table 8). However, it seems that having a superior quality of housing compared with a basic level of housing is not an important job attribute to the average health worker, with an estimated coefficient mean of −0.3 for *house2*.

## Discussion

These DCEs represent the largest DCEs on employment preferences of health workers in post ever undertaken on the African continent. The previous largest experiment of 861 doctors and nurses was by Hanson and Jack [13] undertaken in one country - Ethiopia. The only other cross country study by Blaauw et al. [5] sampled graduating nurses in three countries. Our study contributes to the existing knowledge by focusing on a large cohort of health workers who are engaged in the delivery of emergency obstetric care services. The study was based on a sample of 602 respondents in Malawi, 801 in Tanzania, and 569 in Mozambique, providing a total sample size of 1972. The results are remarkable because of their consistency across the countries. By far the strongest predictors of job choice were shown to be access to continuing professional development and human resources management. The impact of opportunities for career development has been shown continually throughout previous studies, and is usually one of the most important factors underlying job choice. For instance, Mangham and Hanson [27], Blaauw et al. [5], Kolstad [19], Honda & Vio [17] and Takemura et al. [44] all found opportunities for upgrading qualifications and further education to be strong predictors of choice. Human resource management has seldom been captured in previous work. When it has been, studies have shown it to hold predictive power, although somewhat partial definitions have been adopted. Hanson and Jack [13] show the ‘level of supervision’ is one of the most important factors for nurses in Ethiopia, Blaauw et al. [5] report some preference for ‘relational’ over ‘hierarchical’ management style, and Kruk et al. [20] show ‘supportive management style’ to be a key factor for medical students in Ghana. This study uses a more comprehensive definition than previous work –with HRM being described as the overall quality of management, including mechanisms for ‘staff support’, ‘supervision’ and fair and transparent systems of ‘appraisal’. According to our results, the attributes HRM and access to continuing professional development (which is also a component of HRM) hold much more explanatory power than any of the other attributes in our experiments in Tanzania and Mozambique. They also show a high utility in Malawi, though a salary double the base pay level shows higher utility in this sample.

Consistent with similar works we find pay and allowances to be important and significantly positively related to utility, but financial rewards are not as fundamental a factor underlying employment preferences as many may have previously believed. Good human resources management, opportunities for professional development and basic housing are consistently of higher utility than a job that pays one and a half times current base salary. There is evidence to indicate diminishing marginal utility in relation to pay in all three countries. There is emerging observational evidence that pay increases coupled with other initiatives have led to significant improvements in recruitment and retention in Malawi, particularly when this pushes pay above a subsistence level that health workers feel is the minimal acceptable. It may be that once remuneration rates reach a level that allows health workers to meet their basic needs, other considerations become more important than pay. The diminishing marginal utility of pay evident in the results may be an indication that this optimum level is possibly at 1.5 times current basic salaries, as the utility increase is smaller when salary moves from 1.5 to double.

An unexpected finding from this study is the low utility *location* has in job preference. This is contrary to anecdotal evidence that is strongly suggestive of a preference for work in urban rather than rural locations and several studies that have focused on identifying factors that might attract health workers to rural locations (e.g. [5, 22]). There is some evidence in the recent literature that urban locations may not be high priority for all health workers, for example Blaauw et al.’s [5] study found that even in the absence of any human resource policy intervention, 84.2% of recent Thai nursing graduates would choose a rural job, as would 43.4% of the nurse graduates in Kenya. However there continues to be a strong emphasis on incentivising rural postings for health workers.

A limitation of this work as with all DCE results is that it is based on ‘stated preferences’, based on what individuals state they believe, think and will do when presented with hypothetical situations; rather than on ‘revealed preferences’, in terms of choices and behaviour in response to real-life situations. Some might consider the use of an orthogonal fractional factorial, and not an optimal, trial design a limitation of the study but this design was commonly used at the time, although it has since been replaced by more statistically efficient designs. A further limitation of this study is the use of a common comparator. While previously common, this is no longer best practice as it discards much information and can lead to identification problems.

Despite these caveats, DCEs such as this do provide rich and valuable information to guide future policy development, particularly in the context of scarce resources where trade-offs are inevitable and policy priorities need to be more informed by evidence of what is likely to deliver the greatest impact.

## Conclusion

The results are remarkable because of their consistency across the countries. By far the strongest predictors of job choice were shown to be access to continuing professional development and the presence of functioning human resources managemant. Consistent with similar works we find pay and allowances to be important and significantly positively related to utility, but financial rewards are not as fundamental a factor underlying employment preferences as many may have previously believed. There is evidence to indicate diminishing marginal utility in relation to pay in the three countries. Location (urban vs rural) had the smallest effect on utility for job choice in all three countries. These findings are important in the context where efforts to address the human resources crisis have focused primarily on increasing salaries and incentives, as well as providing additional allowances to work in rural areas. Our conclusion is that improving human resources management, and in particular access to continuing professional development, may prove a more effective motivation and retention strategy.

## Appendix 1

### Description of attributes and attribute levels

- Geographic Location
  This attribute specifies whether your place of work is in an urban or rural area.
- Net Monthly Pay (including regular allowances)
  This attribute takes on different levels. The first represents the base salary for a health worker at an “average” grade in the civil service pay scale, while higher levels are multiples of this average base level. Note that the base salary does not necessarily reflect your current actual salary.
- Government-provided Housing
  This attribute measures the existence, and quality, of government-provided housing, and has three possible levels. “None” means there is no housing provided by the government as part of the conditions of employment. “Basic” housing means the government provides housing for the health worker, but that it is rudimentary, having no electricity or running water, and with at best an outside toilet. “Superior” housing means the government provides housing of higher quality, including the presence of electricity and running water, including an inside flush toilet.
- Availability of Equipment and Drugs
  This attribute simply takes on two values – “inadequate” and “improved”. “Inadequate” is the standard of equipment and availability of drugs that you might expect in a poorly equipped public facility in the given location. “Improved” is that level of supplies that would result from a doubling of the budget currently spent on equipment and drugs.
- Access to Continuing Professional Development
  This attribute measures the availability of continuing professional development, in terms of access to further education and upgrading. It has 2 levels - “limited” and “improved”. “Limited’ access means there are very few opportunities, with no clear guidelines on who can avail of them. “Improved” access means there are sufficient opportunities available, with clear policies on the criteria needed to qualify for places.
- Human Resources Management Systems
  This attribute relates to the quality of human resources management in your workplace and it has two values – “poor” and “functioning”. “Poor” describes a management system with either no mechanisms or poorly administered mechanisms for staff support, supervision and appraisal. “Functioning” describes a system where there are transparent, accountable and consistent systems for staff support, supervision and appraisal.

## Appendix 2

### Data collection procedures

- Research Permissions: In each country district/zone, letters or emails were written and sent in advance to the Regional/Zonal/District Medical Officer (DMO) to inform them about the project and the research to be conducted in facilities in their area of responsibility. Before data collection teams visited the selected health care facilities, the teams first introduced themselves to the DMO to receive verbal and/or written permission to proceed with the study. Once permission was granted, teams traveled to each of the selected health care facilities in the region/zone.
- Teams carried with them copies of all communications and permissions obtained to proceed with the research. Teams also carried copies of the Ethical Review permissions received by the project.
- Identification of potential respondents and eligibility criteria: In each facility, teams first introduced themselves and the study to the facility and/or maternity in-charge. They inquired about the number of staff currently working in the maternity unit as well as staff who may have temporarily been assigned to another unit (i.e. out-patient department or the reproductive child health unit). The maternity and/or facility in-charges also helped identify the clinical staff (i.e. doctors, clinical officers and medical assistants) that is called for emergency procedures like caesarean sections. Teams recorded the number of staff in each cadre to ensure that as many of them as possible were approached to participate in the study.
- Teams documented the number of staff in each cadre that were approached to participate in the Provider Survey on a Tally Sheet. The Tally Sheet captured the following information: the cadre of the health worker; the total number of providers approached in each cadre; the total number of eligible respondents (i.e. those who had performed at least one of the EmOC signal functions in the last three months); the total number of refusals, the total number who consented to participate in the Survey; the total number of partially-filled surveys returned; the total number of completed surveys; and the total number of surveys that were not returned.

The Tally Sheets included country-specific lists of cadres to facilitate the documentation process. For Tanzania, the cadres listed included: Medical Officers, Assistant Medical Officers; Clinical Officers; Doctors; Nurses; Medical Attendants/Nursing Attendants and Maternal and Child Health Aides. For Malawi, the cadres listed included: Clinical Officers; Doctors; Nurses/Nurse Midwives—All Levels; and Medical Assistants.

- Once health care providers were introduced to the study, data collectors determined eligibility by showing them a list of the EmOC signal functions to determine whether they had performed at least one of the signal functions in the last three months. Eligible providers received a background of the purpose of the research study and asked if they would be interested in participating in the study. Consent was administered if the provider expressed interest. If providers were not eligible or interested in participating in the study, they were counted on the Tally Sheet under “providers approached.”
- Consent : To administer consent, data collectors briefly summarized the consent information, including the requirements of the study participant. Key points to highlight in administering the consent were provided to each data collector (see Section A below). Providers were then provided with a Consent Information Sheet and asked to sign a Consent Signature Form. Study participants had the option of keeping a copy of the Consent Signature Form.

All signed consent forms were kept in a folder, separate from the completed Provider Surveys in the field. Once data collectors returned from the field, signed consent forms were to be kept in a safe, locked storage space in the country HSSE office.

If respondents refused to sign the consent form or agreed to sign the consent only after completing the survey but then refused to sign the form, they were categorized as “refusals” on the Tally Sheet. If they completed a survey but refused to consent, the survey was returned to the respondent for them to destroy.

- The data collection teams spent up to 2 days at each facility in order to maximize the number of eligible staff approached to participate in the study. Team members were instructed to visit facilities during different shifts. Key points to highlight about the survey were provided to teams to solicit participation.
- The Provider Survey is a self-administered survey. Once consent was obtained from participants, they were handed a copy of the survey form to complete independently and advised to read the instructions for each section carefully before completing. The data collection team informed respondents that they were available to answer any questions or provide clarification if needed. Data collectors informed respondents of how long they would be at the facility and arranged to collect completed surveys.
- As surveys were returned, data collectors quickly reviewed the instruments for completeness with the respondent present. This was done in order to try to maximize completeness and address any clarifications needed for skipped items.
- Identification numbers were assigned to each Provider Survey upon return. Identification numbers were derived using the facility identification number plus two digits. Upon return, a unique identification number was written on every page of a Provider Survey.

## Appendix 3

### The mixed logit model parameter estimation

The mixed logit is a highly flexible discrete choice model that can approximate any random utility model [31]. Hensher and Greene [14] and Train [46] describe this model in detail. The flexibility of the mixed logit results from the fact that it removes some of the restrictive assumptions imposed by conditional or multinomial logit models. One of these includes the assumption of irrelevant alternatives (IIA), which implies that the relative odds of an individual choosing one alternative over another is unchanged by the inclusion or exclusion of other alternatives. This assumption can be unrealistic in certain situations.

Conditional or multinomial logit models also assume that error terms are independently and identically (iid) distributed. This is not always appropriate, particularly in circumstances where there are repeated measurements from respondents (e.g. each individual is presented with multiple choice situations), as individuals’ choices are likely to be correlated over choice sets. The mixed logit model allows for variation in the tastes of individuals in the population. It achieves this by including random coefficients for each individual that induce correlations between the unobserved utilities over repeated choices of each individual [35].

Observed responses from participants are of the form

1 $$ {y}_{ijt}=\left\{\begin{array}{cc}\hfill 1\hfill & \hfill if\  observation\ i\  chooses\  alternative\ j\  in\  choice\  situation\ t\hfill \\ {}\hfill 0\hfill & \hfill otherwise.\hfill \end{array}\right. $$

In the mixed logit model, the utility that person i derives from alternative j in choice situation t can be represented by

2 $$ {U}_{ijk}={\beta_i}^{\prime }{x}_{ijt}+{e}_{ijt} $$

where x_ijt_ are observed variables representing attributes specific to alternative j in choice situation t presented to individual i, while ε_ijt_ is an error term that is an IID extreme value type I deviate. The parameter β_i_ is a vector of random coefficients representing the effects of variables in x_ijt_ on person i’s utility (the tastes of individual i). Note that in a standard (multinomial or conditional) logit model, the coefficient vector is assumed to be constant across individuals and is therefore not indexed by i.

The random coefficient vector may be decomposed into a systematic component that varies with observed individual-specific variables z_i_ (such as socio-demographic variables observed for each individual i), and a stochastic component *η*_*i*_ that varies randomly across individuals.

3 $$ {\beta}_i=\varOmega {z}_i+{\eta}_i $$

The mixed logit model assumes a general class of distributions for *η*_*i*_ (such as the normal, lognormal, uniform, or triangular distributions). The systematic component *Ωz*_*i*_ allows for heterogeneity around the mean of this distribution that can be attributed to the observed individual-specific variables *z*_*i*_. Except when this distribution is the lognormal, this is equivalent to including fixed effect interaction terms between the alternative-specific attributes, *x*_*ijt*,_ and the individual-specific characteristics, *z*_*i*_, in the utility function [14].

The underpinning assumption of the discrete choice model is that individual i chooses alternative j in choice situation t if it results in the maximum utility from the set of alternatives in situation t; that is to say, an individual chooses alternative j in choice situation t if and only if

4 $$ {U}_{ijt}>{U}_{ikt};\forall k\ne j $$

Since utility is unobserved, it is only differences in utility that are relevant in the analysis of discrete choice data. Individuals choose between J alternatives in each choice situation, so that j = {1,2,…,J}. It is of interest to model the probability of an individual choosing each alternative over the others in a given choice situation. In a mixed logit model for discrete choice data, the probability of individual i choosing alternative j in choice situation t, (conditional on η_i_) is

5 $$ \mathrm{P}\left({\mathrm{y}}_{\mathrm{i}\mathrm{jt}}=1\Big|{\mathrm{X}}_{\mathrm{i},\mathrm{t}},\ {\mathrm{z}}_{\mathrm{i}},\ \Omega,\ {\upeta}_{\mathrm{i}}\right)=\mathrm{P}\left(\ {U}_{ijt}\ge\ {U}_{ikt}\ :k=\left\{1,\dots, J\right\}\right) = \frac{ \exp\ \left\{{\left({\Omega \mathrm{z}}_{\mathrm{i}} + {\upeta}_{\mathrm{i}}\right)}^{\prime }{\mathrm{x}}_{\mathrm{i}\mathrm{jt}}\right\}}{{\displaystyle {\sum}_{\mathrm{k}}} \exp \left\{{\left({\Omega \mathrm{z}}_{\mathrm{i}} + {\upeta}_{\mathrm{i}}\right)}^{\prime }{\mathrm{x}}_{\mathrm{i}\mathrm{kt}}\right\}} $$

where X_i,t_ = {x_i1t_,…, x_iJt_} is the set of attributes for each alternative in choice situation t.

### Parameter estimation

The parameters of a mixed logit model are more difficult to estimate than those of a standard logit model, due to the random coefficients that enter the utility function.

Let Y_i_ = {y_ijt_: j = 1,…,J; t = 1,…,T} denote the sequence of choices made by respondent i over T choice situations and let X_i_ = {x_ijt_: j = 1,…,J; t = 1,…,T} be vectors of alternative-specific attributes for each alternative j within each choice situation t. Also, let I(i,t) indicate the alternative chosen by individual i in choice situation t. Then, the probability of the sequence of choices made by respondent i over choice situations t = 1,2,…T, conditional on that individual’s tastes β_i_, is

6 $$ \mathrm{P}\left({\mathrm{y}}_{\mathrm{i}}\ \Big|{\mathrm{X}}_{\mathrm{i}},\ {\mathrm{z}}_{\mathrm{i}},\ \Omega,\ {\upeta}_{\mathrm{i}}\right) = {\displaystyle {\prod}_{\mathrm{t}=1}^{\mathrm{T}}}\mathrm{P}\left({\mathrm{y}}_{\mathrm{i}\mathrm{I}\left(\mathrm{i},\mathrm{t}\right)\mathrm{t}}\ \Big|{\mathrm{X}}_{\mathrm{i},\mathrm{t}},\ {\mathrm{z}}_{\mathrm{i}},\ \Omega,\ {\upeta}_{\mathrm{i}}\right) = {\displaystyle {\prod}_{\mathrm{t}=1}^{\mathrm{T}}}\frac{ \exp \left\{\left({\Omega \mathrm{z}}_{\mathrm{i}} + {\upeta}_{\mathrm{i}}\right)^{\prime }\ {\mathrm{x}}_{\mathrm{i}\mathrm{I}\left(\mathrm{i},\mathrm{t}\right)\mathrm{t}}\right\}}{{\displaystyle {\sum}_{\mathrm{k}}} \exp \left\{\left({\Omega \mathrm{z}}_{\mathrm{i}} + {\upeta}_{\mathrm{i}}\right)^{\prime }\ {\mathrm{x}}_{\mathrm{i}\mathrm{kt}}\right\}} $$

The random effect η_i_ varies over the population with density f(*η*_i_| θ), where *θ* is the set of distributional parameters (e.g. for the normal distribution, it would be the mean μ and covariance ∑). Therefore, the unconditional probability of an individual’s observed choices is obtained by integration over this density

7 $$ \mathrm{P}\left({\mathrm{y}}_{\mathrm{i}}\ \Big|{\mathrm{X}}_{\mathrm{i}},\ {\mathrm{z}}_{\mathrm{i}},\ \Omega,\ \uptheta \right) = {\displaystyle \int}\mathrm{P}\left({\mathrm{y}}_{\mathrm{i}}\ \Big|{\mathrm{X}}_{\mathrm{i}},\ {\mathrm{z}}_{\mathrm{i}},\ \Omega,\ {\upeta}_{\mathrm{i}}\right)\ \mathrm{f}\left({\upeta}_{\mathrm{i}}\ \Big|\uptheta \right)\ {\mathrm{d}\upeta}_{\mathrm{i}}. $$

The log likelihood of the mixed logit is

8 $$ LL\left(\theta,\ \Omega \right) = {\displaystyle \sum_{i=1}^N} \ln \mathrm{P}\left({\mathrm{y}}_{\mathrm{i}}\ \Big|{\mathrm{X}}_{\mathrm{i}},\ {\mathrm{z}}_{\mathrm{i}},\ \Omega,\ \uptheta \right) $$

where N is the number of individuals in the sample. Maximum likelihood estimates must be approximated numerically, due to open form of the log likelihood function.

Mixed logit models were fitted here using the *mixlogit* command [16] in Stata Version 12.1. Estimation is computationally intensive as it requires numerical integration over the distribution of random parameters, and is carried out using maximum simulated likelihood methods. Further details on mixed logitestimation procedures may be found in Hensher and Greene [14], Train [46] and Hole [16]. The *mixlogit* function allows the specification of correlated random coefficients. For each mixed logit model, likelihood ratio tests were carried out to test for the inclusion of correlated (vs. independent) random effects. In all cases, likelihood ratio tests produced statistically significant *p*-values. Therefore, correlated random coefficients have been included in all mixed logit models.
